# Prevalence of proximate risk factors of active tuberculosis in latent tuberculosis infection: A cross-sectional study from South India

**DOI:** 10.3389/fpubh.2022.1011388

**Published:** 2022-10-06

**Authors:** Saravanan Munisankar, Anuradha Rajamanickam, Suganthi Balasubramanian, Satishwaran Muthusamy, Pradeep Aravindan Menon, Shaik Fayaz Ahamed, Christopher Whalen, Paschaline Gumne, Inderdeep Kaur, Varma Nadimpalli, Akshay Deverakonda, Zhenhao Chen, John David Otto, Tesfalidet Habitegiyorgis, Harish Kandaswamy, Subash Babu

**Affiliations:** ^1^National Institutes of Health-National Institute for Research in Tuberculosis-International Center for Excellence in Research, Chennai, India; ^2^National Institute for Research in Tuberculosis, Chennai, India; ^3^Office of Cyber Infrastructure and Computational Biology, National Institute of Allergy and Infectious Diseases, National Institutes of Health, Bethesda, MD, United States; ^4^Laboratory of Parasitic Diseases, National Institutes of Allergy and Infectious Diseases, National Institutes of Health, Bethesda, MD, United States

**Keywords:** latent tuberculosis, diabetes mellitus, hypertension, undernutrition, obesity, co-morbidity

## Abstract

The prevalence of proximate risk factors for active tuberculosis (TB) in areas of high prevalence of latent tuberculosis infection (LTBI) is not clearly understood. We aimed at assessing the prevalence of non-communicable multi-morbidity focusing on diabetes mellitus (DM), malnutrition, and hypertension (HTN) as common risk factors of LTBI progressing to active TB. In a cross-sectional study, 2,351 adults (45% male and 55% female) from villages in the Kancheepuram district of South India were enrolled between 2013 and 2020. DM was defined as HbA1c >6.4%, undernutrition was defined as low body mass index (LBMI) <18.5 kg/m^2^, obesity was classified as BMI ≥25 kg/m^2^, HTN was reported as systolic pressure >130 mmHg, and LTBI was defined as positive (≥ 0.35 international units/ml) by QuantiFERON Gold In-Tube assay. A total of 1,226 individuals (52%) were positive for LTBI out of 2351 tested individuals. The prevalence of DM and pre-diabetes mellitus (PDM) was 21 and 35%, respectively, HTN was 15% in latent tuberculosis (LTB)-infected individuals. The association of DM [odds ratio (OR)]; adjusted odds ratio (aOR) (OR = 1.26, 95% CI: 1.13–1.65; aOR = 1.19, 95% CI: 1.10–1.58), PDM (OR = 1.11, 95% CI: 1.0–1.35), and HTN (OR = 1.28, 95% CI: 1.11–1.62; aOR = 1.18, 95% CI: 1.0–1.56) poses as risk factors of LTBI progression to active TB. The prevalence of LBMI 9% (OR = 1.07, 95% CI: 0.78–1.48) and obesity 42% (OR = 0.85, 95% CI: 0.70–1.03) did not show any statistically significant association with LTB-infected individuals. The present evidence of a high burden of multi-morbidity suggests that proximate risk factors of active TB in LTBI can be managed by nutrition and lifestyle modification.

## Introduction

Tuberculosis (TB) has been a major health challenge across the globe, affecting around 10 million people, of which 2.6 million are in India, the country with the world's largest TB burden (1). An estimated 1.7 billion people forming 23% of the world's population have been reported to have latent tuberculosis infection (LTBI). LTBI progression to TB is estimated at a risk rate of 10% (2). It is approximated that about 26% of the Indian population have been victimized with TB bacteria, the majority of them having latent tuberculosis (LTB) rather than the active form of TB disease (1). An estimated 537 million adults are living with diabetes mellitus (DM), ~74 million of whom are in India. This DM burden is the second largest globally and is expected to rise to 783 million by 2045 (3). Clinical and epidemiological studies in the past have pointed out DM increases the risk of developing active TB, and though the association between DM and active TB has been well documented (4), data elucidating the relationship between DM and LTBI are scarce. The weakened immunity in individuals with DM could be a possible reason, thereby facilitating either TB infection or LTB reactivation (5).

Undernutrition is defined as low body mass index (LBMI) and TB have a bidirectional relationship. LBMI elevates the risk of developing active TB by about six- to 10-fold. One-quarter of TB in the world results from malnutrition, the risk of which can be decreased by improving the nutritional profile of the individual (6). Metabolic syndrome is diagnosed based on DM, hypertension (HTN), and obesity. In prospective investigations done over the years, midlife obesity and midlife HTN have been found to increase the risk of later impairment (7). Epidemiological investigations carried out early have failed to look into the fact that DM, obesity, and HTN tend to combine in individuals and are highly associated (8).

Therefore, we conducted a cross-sectional study on the prevalence of the most commonly reported risk factors for TB in LTB-infected individuals. A quantitative assessment was performed on non-communicable multi-morbidity focusing on DM, LBMI, obesity, and HTN as common risk factors of LTBI progressing to active TB.

## Methods

### Ethics statement

The study was approved by the Institutional Review Boards of the National Institute of Allergy and Infectious Diseases (USA) and National Institute for Research in Tuberculosis (NIRT-IEC-2011 013), Chennai, in adherence to all ethical considerations, and informed written consent was obtained from all participants.

### Study design

We performed a cross-sectional study to identify the prevalence of proximate risk factors for active TB in areas of high prevalence of LTBI.

### Study population

Around 4,500 individuals were screened for tuberculin skin test (TST), and positive individuals were included in the study. An assumption was made that the LTB prevalence in the general population is 50% based on previous data (9). We took a random sample of size 4,500 from the population. The study had all consenting individuals (18–65 years of age) enrolled from 2013 to 2020 from the villages spanning an area of 5 km radius in the Kancheepuram district of South India. Agriculture was predominant in this region. Low income and lack of education among the community members, who are daily wage laborers, have been observed in all screened regions. However, the basic necessities of food and water supply were met. Anthropometric measurements (height, weight, and waist circumference), then biochemical parameters, HbA1c (glycated hemoglobin) level, and blood pressure were procured using standardized techniques. Individuals with symptoms or signs of active TB, history of previous TB, known cases of cancer, human immunodeficiency virus (HIV), or other immunosuppressive illness, and TST performed within the last 6 months prior to screening were considered in the exclusion criteria. Household Global Positioning System (GPS) coordinates of all the eligible individuals were also collected.

### Data variables

The primary outcomes of interest as co-morbidity were DM or pre-diabetes mellitus (PDM) defined on the basis of HbA1c percentages, using American Diabetes Association criteria (DM, >6.4%; PDM, 5.7–6.4%) using HbA1C kit (Beckman Coulter, Clare, Ireland) (10), LBMI was described based on the American Heart Association/American College of Cardiology guidelines (LBMI ≤ 18.5 kg/m^2^), and measuring serum albumin <3.4 g/dl (Beckman Coulter, Clare, Ireland) at fasting stage, overweight by body mass index (BMI) 23–24.9 kg/m^2^, and obesity defined by BMI threshold of ≥25.0 kg/m^2^. HTN was reported as systolic pressure >130 mmHg based on American Heart Association guidelines (11). LTBI status was diagnosed based on TST using two tuberculin units of Tuberculin PPD RT 23 SSI (Serum Statens Institute, Denmark). A positive skin test was defined as an induration of at least 12 mm diameter and then by QuantiFERON-TB Gold in-tube (QFT-GIT) assay with the positivity (≥0.35 international units**/**ml) by going through the QFT-GIT (Qiagen, Maryland, USA) kit instructions, and optical density (OD) was read at 450 nm using Spectramaxi3X (Molecular devices), and in addition, socio-demographic characteristics such as age and sex were analyzed.

### Data collection and management

Paper-based, standardized and structured case reporting forms and e-data capture methods (miForms, REDCap) were used for data collection by trained study staff, and the iDatafax clinical data management system was used for secure data management of patient identifiers, demographic, laboratory, and clinical data. Maps were made in QGIS 3.10.11; study data were displayed after processing in PostgreSQL and Pentaho. Information, from OpenStreetMap and the OpenStreetMap Foundation, was used through an Open Database License. OpenStreetMap data were styled according to guidelines by https://github.com/charlesmillet.

### Data analysis

Statistical analyses were carried out to estimate the prevalence of the most commonly reported risk factors for TB in LTB-infected individuals focusing on DM, LBMI, and HTN. Statistical results were based on two-sided tests, and the *p*-value ≤ 0.05 was considered statistically significant. Descriptive analysis was done for basic socio-demographic factors and clinical characteristics of LTBI individuals based on QFT status. Mann–Whitney test was done for comparison of the two groups. Categorical variables were compared using the chi-squared test. Logistic regression analysis was done to assess the association between LTBI and potential covariates. *P*-value ≤ 0.05 was taken into consideration as statistical significance. A binomial test was done to determine the prevalence odds ratio by using Clopper–Pearson exact test with 95% CI. Data were analyzed using IBM-SPSS package version 25. REDCap electronic data capture tools were used to collect and manage data, hosted at National Institute for Research in Tuberculosis–International Center for Excellence in Research (ICMR-NIRT-NIH-ICER), Chennai, which provides (1) an intuitive interface for validated data capture; (2) audit trails for tracking data manipulation and export procedures; (3) automated export procedures for seamless data downloads to common statistical packages; and (4) procedures for data integration and interoperability with external sources (12, 13).

## Results

### Socio-demographic and clinical characteristics

Of 2,351 TST-positive adults enrolled in this study, 1,226 adults (52%) were positive for both TST and QFT-Plus and so have been considered positive for LTBI (Table 1). The median age of LTB-infected adults was 40 years (Interquartile Range [IQR] 32–50). Male exhibit adjusted odds ratio (aOR) 1.49, 95% CI: 1.26–1.76 as higher odds of LTBI (Table 2). Age ≥55 years showed a significant association with LTBI odds ratio (OR) 1.46, 95% CI: 1.13–1.89 and (aOR = 1.33, 95% CI: 1.1–1.76) (Table 2). The prevalence of non-communicable outcomes of interest as co-morbidity was DM 21% (256/1,226), HTN 15% (185/1,226), LBMI 9% (108/1,226), and obesity 42% (521/1,226) (Table 1). Age-wise distribution among all villages given with their respective percentages against the total screened subjects can be found in Supplementary Figure 1.

**Table 1:** Socio-demographic and clinical characteristics of latent tuberculosis-infected South Indian adult population enrolled between 2013 and 2020.

**Variable**	**Total n (%)**	**LTB positive** **n (%)** **GM (range)**	**LTB negative** **n (%)** **GM (range)**	**P-value**
Total, n	2,351 (100)	1,226 (52)	1,125 (48)	
**Socio-demographic characteristics-sex**				
Female	1,282 (55)	612 (50)	670 (60)	0.99
Male	1,069 (45)	614 (50)	455 (40)	
**Age, years (median)**	
18–34 (28)	793 (34)	381 (31)	412 (37)	0.0002
35–44 (39)	671 (29)	355 (29)	316 (28)	
45–54 (49)	543 (23)	292 (24)	251 (22)	
≥55 (58)	344 (14)	198 (16)	146 (13)	
**BMI (kg/m** ^**2**^ **)**	
Normal (18.5–22.9)	697 (30)	379 (31) 20.9 (18.5–22.9)	318 (28) 20.9 (18.5–22.9)	0.89
Undernourished ( ≤ 18.5)	192 (8)	108 (9) 17 (12.3–18.5)	84 (7) 18.8 (14.0–18.5)	
Overweight (23.0–24.9)	430 (18)	218 (18) 23.9 (23–24.9)	212 (19) 24.0 (23.0–24.9)	
Obesity (≥25.0)	1,032 (44)	521 (42) 28.4 (25–41.5)	511 (45) 28.6 (25.0–43.8)	
**HbA1c (%)**	
NDM ( ≤ 5.7)	1,104 (47)	551 (45) 5.3 (3.6–5.7)	553 (49) 5.3 (3.9–5.7)	0.03
PDM (>5.7– ≤ 6.4)	788 (33)	429 (35) 6 (5.7–6.4)	359 (32) 5.9 (5.7–6.4)	
DM (>6.4)	459 (20)	256 (21) 7.9 (6.4–18.4)	203 (18) 8.0 (6.4–16.0)	
**HTN (mm Hg)**	
Systolic pressure ≤ 130	2,029 (86)	1,041 (85) 113 (100–130)	988 (88) 112 (100–130)	0.41
Systolic pressure >130	322 (14)	185 (15) 150 (132–2000)	137 (12) 148.6 (132–200)	

LTB, latent tuberculosis; BMI, body mass index; HbA1c, glycated hemoglobin; NDM, non-diabetes mellitus; PDM, pre-diabetes mellitus; DM, diabetes mellitus; HTN, hypertension; and GM, geometric mean.

**Table 2:** Association of clinical co-morbidities with latent tuberculosis-infected South Indian adult population enrolled between 2013 and 2020.

**Variable**	**LTBI**	**P-value**	**LTBI**	**P-value**
	**OR (95% Cl)**		**aOR (95% Cl)**	
**Socio-demographic characteristics-sex**				
Female	Reference	0.001	Reference	0.001
Male	1.47 (1.25–1.74)		1.49 (1.26–1.76)	
**Age, years**				
18–34	Reference	0.018	Reference	0.043
35–44	1.21 (0.98–1.49)		1.23 (0.99–1.52)	
45–54	1.25 (1.01–1.56)		1.24 (0.98–1.57)	
≥55	1.46 (1.13–1.89)		1.33 (1.1–1.76)	
**BMI (kg/m** ^**2**^ **)**				
Normal (18.5–22.9)	Reference	0.243	Reference	0.389
Undernutrition ( ≤ 18.5)	1.07 (0.78–1.48)		1.15 (0.83–1.60)	
Overweight (23.0–24.9)	0.86 (0.67–1.09)		0.82 (0.64–1.05)	
Obesity (≥25.0)	0.85 (0.70–1.03)		0.84 (0.69–1.03)	
**HbA1c (%)**				
NDM ( ≤ 5.7)	Reference	0.032	Reference	0.040
PDM (>5.7– < 6.4)	1.11 (1.0–1.35)		1.10 (0.90–1.35)	
DM (>6.4)	1.26 (1.13–1.65)		1.19 (1.10–1.58)	
**HTN (mm Hg)**
Systolic pressure ≤ 130	Reference	0.028	Reference	0.043
Systolic pressure >130	1.28 (1.11–1.62)		1.18 (1.0–1.56)	

LTB, latent tuberculosis; BMI, body mass index; HbA1c, glycated hemoglobin; NDM, non-diabetes mellitus; PDM, pre-diabetes mellitus; DM, diabetes mellitus; HTN, hypertension; Cl, confidence interval; OR, odds ratio; and aOR, adjusted odds ratio.

### Geographical distribution of LTBI

**Figure 1:**
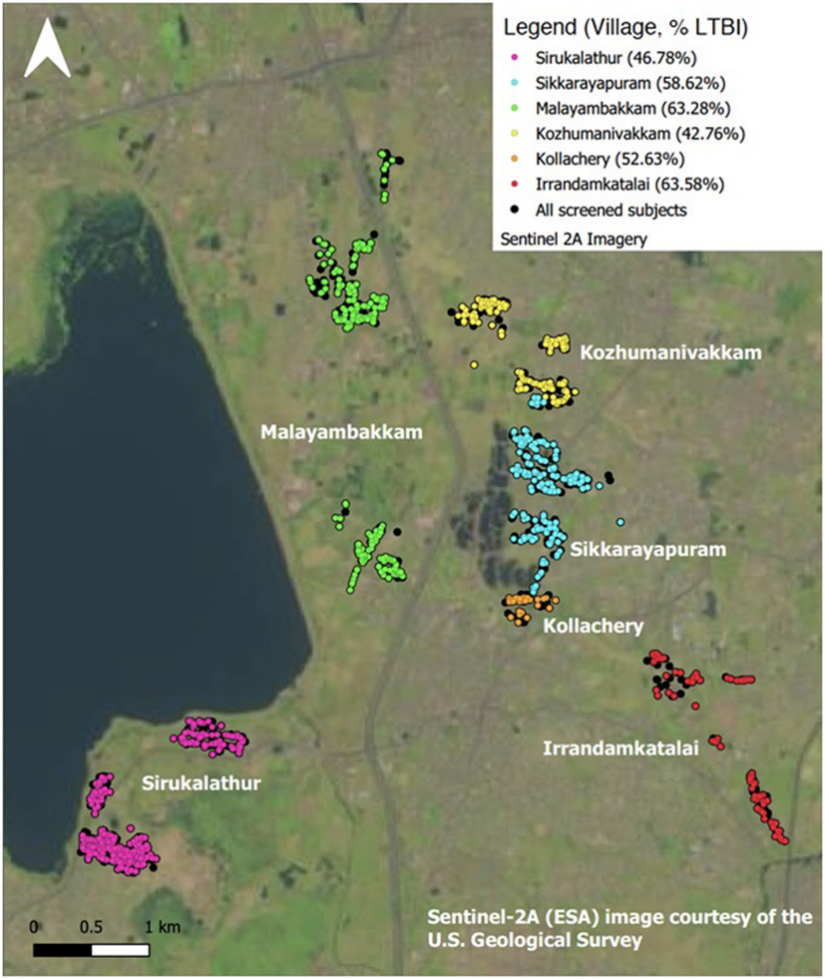
Distribution of LTBI by study site. Map made in QGIS 3.10.11, study data displayed after processing in PostgreSQL and Pentaho. Contains information from OpenStreetMap and OpenStreetMap Foundation, which is made available under the Open Database License. OpenStreetMap data styled according to guidelines by https://github.com/charlesmillet.

Our prevalence study was conducted in six villages of Kancheepuram district in South India. The following prevalence rates were observed in the villages—Kollacherry 52.63% (30/57), Sikkarayapuram 58.62% (221/377), Sirukalathur 46.78% (407/870), Malayambakkam 63.28% (274/433), Kozhumanivakkam 42.76% (198/463), and Irandamkattalai 63.58% (96/151). The prevalence of LTBI in each village surveyed is depicted as a map (Figure 1), and the size of LTBI clusters was taken at 5 km radius (Supplementary Figure 2).

### Prevalence and association of DM and PDM with LTBI

**Figure 2:**
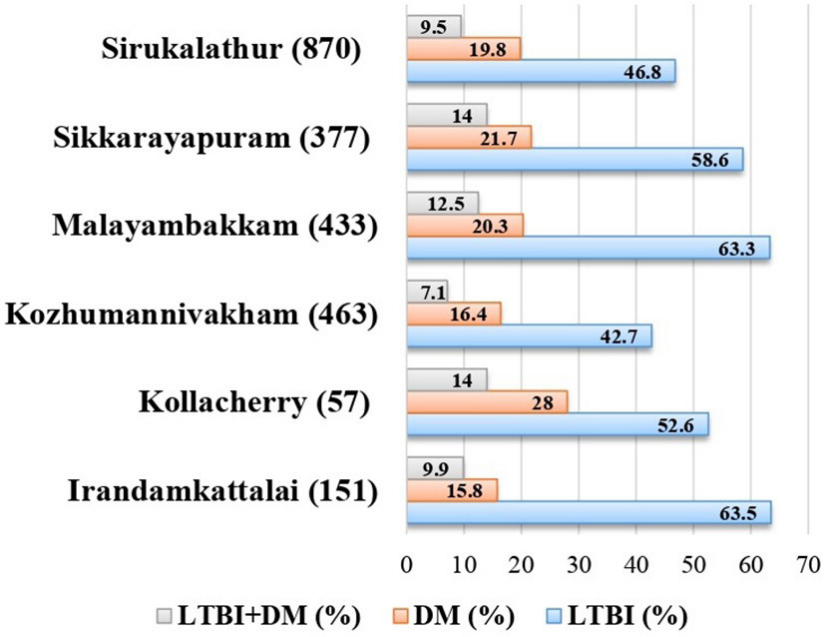
Prevalence of LTBI and DM village wise. LTBI, latent tuberculosis infection; DM, diabetes mellitus.

The median HbA1c among LTBI with DM and PDM was, respectively, 6.9% (IQR 6.4–8.4) and 5.9% (IQR 5.8–6.1). There was a significant association between DM (OR = 1.26, 95% CI 1.13–1.65), PDM (OR = 1.11, 95% CI 1.0–1.35), and prevalent LTBI (Table 2). The highest percentage of DM was seen in Kollacherry 28% (16/57). Kollacherry and Sikkarayapuram recorded the highest percentage of LTBI+DM cases 14% (8/57) (Figure 2). Village-wise distribution and classification of DM are given in Supplementary Figure 3.

### Prevalence and association of BMI with LTBI

Among 1,226 LTBI individuals with BMI measurements, 18% (218/1,226) participants were overweight, 42% (521/1,226) were obese, and 9% (108/1,226) were LBMI (Table 1) (Supplementary Figure 4). There was no association observed between BMI and LTBI in both unadjusted and adjusted odds fashions (Table 2).

### Prevalence and association of HTN with LTBI

**Figure 3:**
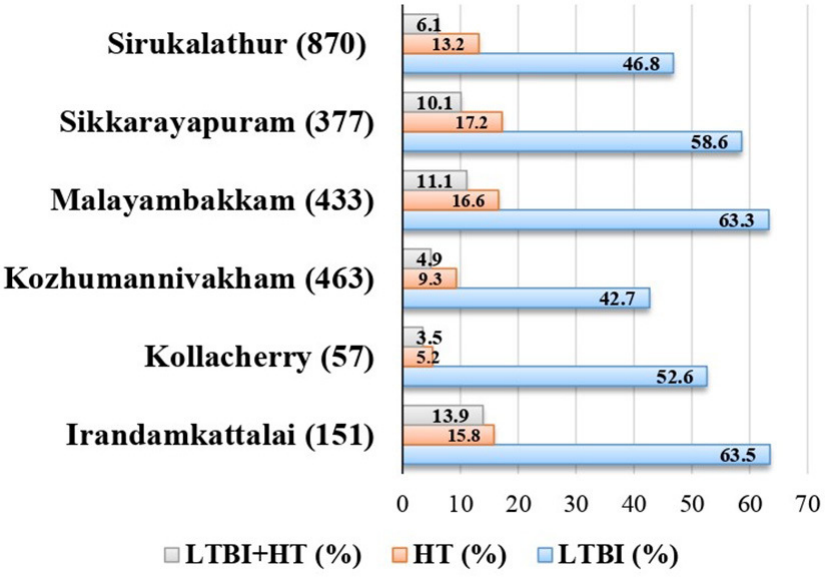
Prevalence of LTBI and HTN village wise. LTBI, latent tuberculosis infection; HTN, hypertension.

HTN exhibited a significant association with LTBI (OR = 1.28, 95% CI 1.11–1.62). About 57.4% (124/216) of prevalence for HTN is seen in LTBI individuals above the age of 45. Sikkarayapuram had the highest percentage of individuals with HTN 17.2% (65/377). LTBI+HTN cases were maximum in Irandamkattalai 13.9% (21/151) (Figure 3). The prevalence and classification of HTN in LTB-infected individuals screened across six villages are given in Supplementary Figure 5.

### Proximate risk factors of TB in LTBI

The study population had at least one non-communicable co-morbidity. The most common were DM 21% (256/1,226), PDM 35% (429/1,226), LBMI 9% (108/1,226), and HTN 15% (185/1,226). The association was determined by the inclusion of potential confounders in the regression models: adjusted for age, sex, BMI, DM, and HTN. However, unfavorable outcomes were more likely with DM (aOR=1.19, 95% CI 1.10–1.58, *p* < 0.040) and HTN (aOR=1.18, 95% CI 1.0–1.56, *p* < 0.043) (Table 2). In addition, HTN showed a significant association with DM in prevalence odds ratio (POR) (2.95; 95% CI, 2.26–4.12; *p* < 0.0001).

## Discussion

We present findings on the prevalence and association of multi-morbid factors in LTB-infected individuals in a rural setting of South India. The overall prevalence of LTB positivity 52% (1,226/2,351) was estimated using QFT test in TST-positive individuals between 2013 and 2020. The US Centers for Disease Control and Prevention and the Canadian Tuberculosis Committee recommend dual testing using TST and an IGRA (14), TST being highly sensitive and less specific, while IGRA test is more specific and sensitive compared to TST. In our study area due to the high prevalence of atypical mycobacteria, TST positivity is not as reliable as QFT positivity. We found high prevalence of LTBI in Irandamkattalai 63.5% (96/151) and Malayambakkam 63.3% (274/433), which are poor rural areas with minimal awareness with regards to the spread. Household contacts are major representations of areas with the severe spread of *M. tuberculosis*. However, the effect of such an occurrence on the overall burden of disease at the community level remains unknown. An estimate of community spread from studies from South Africa is responsible for >80% (15). Less than 1% of households in a community are affected by TB disease at any time. The probability of exposure of a TB-infected individual with their social network is numerous. In this accord, it has been shown that the population credited fraction of household exposure was < 20% (16). The participants in this study, who are daily wage laborers, have high chances of exposure in the community leading to the transmission of the disease. The non-communicable co-morbidities such as DM, BMI, and HTN were focused on LTBI. Our study shows that the prevalence of multi-morbidity with higher odds is associated significantly (*p* < 0.001) in males.

A meta-analysis including 12 cross-sectional studies showed DM is associated significantly as a risk factor for LTBI (17–20). Previous observational studies revealed that diabetics were 3.1 times prone to have TB than non-diabetics (21). Lee et al. showed age as important confounding factor and associated with both DM and LTBI. The prevalence of DM and PDM in our study was observed as 56 and 54%, respectively, in LTBI individuals; in addition, we reported that the crude OR (1.26; 95% CI, 1.13–1.65) was substantially larger than the aOR (1.19; 95% CI, 1.10–1.58). Our study therefore supports the existence of an increased risk of LTBI, though magnitude of the same cannot be ascertained numerically. Cohort studies showed that DM is linked to a two- to three-fold increase in TB risk (17) as this non-communicable disease has the potential to decrease host immunity and further leads to increased vulnerability to TB infection (22).

Of the many case reports and controlled studies, a few reported that a significant variations in the presence of HTN between TB patients and non-TB controls (23), but HTN did not reveal association or a direct factor of risk for the progression of active TB infection, except in the case of the renal TB (24). HTN is suggested to have a subtle role on the immune system, (25) thereby increasing the risk of TB. TB might also be related indirectly to HTN through DM, which in turn is strongly associated with risk of Cardio Vascular Disease (CVD) (26). In our study, HTN showed a significant association with LTBI in unadjusted odds analysis (*p* < 0.041) and in adjusted odds analysis (*p* < 0.041). There is a notable overlap between DM and HTN, showing an overlap in their etiology and mechanisms of the disease. Landsberg and Molitch (27) stated that in US population, the occurrence of HTN is approximately 30% in type 1 DM individuals and 50 to 80% in type 2 DM individuals. A cohort study documented that type 2 DM was 2.5 times prone to develop in individuals with HTN (28). HTN and DM combinedly resulted in metabolic syndrome. In this study, HTN showed a significant association with DM in POR (2.95; 95% CI, 2.26–4.12; *p* < 0.0001) with 14% (107/781) as rate of prevalence. The combined effect of DM and HTN possibly remains as confounders in showing association with LTBI. Therefore, optimization of lifestyle remains the cornerstone in the prevention and treatment of DM and HTN. This in turn could help in drastically reducing the risk of LTBI and its further progression to active TB infection, especially in a TB-prone population in India. Limitations in this study include less socio-demographics data collection and the need to follow-up in identifying LTB-infected individuals who have progressed to active TB.

## Conclusion

Our study indicated the prevalence of relatively high burden of LTBI in the rural settings of South India. DM, PDM, and HTN were commonly observed proximate risk factors of active TB in LTBI. The existing trend needs management, protection, and lifestyle modification.

## Data availability statement

The original contributions presented in the study are included in the article/Supplementary material, further inquiries can be directed to the corresponding author/s.

## Ethics statement

The studies involving human participants were reviewed and approved by Institutional Review Boards of the National Institute of Allergy and Infectious Diseases (USA) and National Institute for Research in Tuberculosis (NIRT-IEC-2011 013), Chennai. The patients/participants provided their written informed consent to participate in this study.

## Author contributions

All authors listed have made a substantial, direct, and intellectual contribution to the work and approved it for publication.

## Funding

This work was funded by the Division of Intramural Research (DIR), NIAID, and NIH. The funders had no role in the study design, collection of data, analysis and interpretation of data, writing of the manuscript, or decision to submit for publication.

## Conflict of interest

The authors declare that the research was conducted in the absence of any commercial or financial relationships that could be construed as a potential conflict of interest.

## Publisher's note

All claims expressed in this article are solely those of the authors and do not necessarily represent those of their affiliated organizations, or those of the publisher, the editors and the reviewers. Any product that may be evaluated in this article, or claim that may be made by its manufacturer, is not guaranteed or endorsed by the publisher.

## Supplementary material

Supplementary Figure S1 Age-wise distribution of LTB-infected individuals. Age-wise distribution of LTB-infected individuals screened across six villages.

Supplementary Figure S2 Radius of villages screened for the prevalence of LTBI. Prevalence of LTBI screened in six villages of Kancheepuram district with 5 km radius from the center point.

Supplementary Figure S3 Prevalence of diabetes. Prevalence and classification of diabetes mellitus in LTB-infected individuals screened across six villages.

Supplementary Figure S4 Prevalence of BMI. Prevalence and classification of BMI in LTB-infected individuals screened across six villages.

Supplementary Figure S5 Prevalence of HTN. Prevalence and classification of HTN in LTB-infected individuals screened across six villages.

## References

[1] World Health Organization. Global report on Tuberculosis. Geneva, Switzerland: WHO (2021).

[2] World Health Organization. Global Tuberculosis Report. Geneva: (WHO) (2020).

[3] International Diabetes Federation. IDF Diabetes Atlas, Tenth Edition: IDF. Belgium (Virtual) (2021).

[4] Leung CC Lam TH Chan WM Yew WW Ho KS Leung GM. Diabetic control and risk of tuberculosis: a cohort study. Am J Epidemiol. (2008) 167:1486–94. 10.1093/aje/kwn075 18400769

[5] Kumar Nathella P Babu S. Influence of diabetes mellitus on immunity to human tuberculosis. Immunology. (2017) 152:13–24. 10.1111/imm.12762 28543817 PMC5543489

[6] Feleke BE Feleke TE Biadglegne F. Nutritional status of tuberculosis patients, a comparative cross-sectional study. BMC Pulm Med. (2019) 19:182. 10.1186/s12890-019-0953-0 31638950 PMC6802320

[7] Pedditizi E Peters R Beckett N. The risk of overweight/obesity in midlife and late life for the development of dementia: a systematic review and meta-analysis of longitudinal studies. Age Aging. (2016) 45:14–21. 10.1093/aging/afv151 26764391

[8] Alberti KG Eckel RH Grundy SM Zimmet PZ Cleeman JI Donato KA. Harmonizing the metabolic syndrome: a joint interim statement of the International Diabetes Federation Task Force on Epidemiology and Prevention; National Heart, Lung, and Blood Institute; American Heart Association; World Heart Federation; International Atherosclerosis Society; and International Association for the Study of Obesity. Circulation. (2009) 120:1640–5. 10.1161/CIRCULATIONAHA.109.192644 19805654

[9] Joshi R Narang U Zwerling A Jain D Jain V Kalantri S. Predictive value of latent tuberculosis tests in Indian healthcare workers: a cohort study. Eur Respir J. (2011) 38:1475–7. 10.1183/09031936.00014611 22130764

[10] American Diabetes Association. Practice Guidelines Resources (2022). Available online at: https://diabetes.org/diabetes/a1c (accessed September 09, 2022).

[11] American Heart Association/American College of Cardiology guidelines (2017). Available online at: https://www.acc.org/latest-in-cardiology/ten-points-to-remember/2017/11/09/11/41/2017-guideline-for-high-blood-pressure-in-adults (accessed September 09, 2022).

[12] Harris PA Taylor R Thielke R Payne J Gonzalez N Conde JG. Research electronic data capture (REDCap) – A metadata-driven methodology and workflow process for providing translational research informatics support. J Biomed Inform. (2009) 42:377–81. 10.1016/j.jbi.2008.08.010 18929686 PMC2700030

[13] Harris PA Taylor R Minor BL Elliott V Fernandez M O'Neal L. REDCap Consortium, The REDCap consortium: building an international community of software partners. J Biomed Inform. (2019) 95:103208. 10.1016/j.jbi.2019.103208 31078660 PMC7254481

[14] Elzi L Steffen I Furrer H Fehr J Cavassini M Hirschel B. Improved sensitivity of an interferongamma release assay (T-SPOTTB™) in combination with tuberculin skin test for the diagnosis of latent tuberculosis in the presence of HIV co-infection. BMC Infect Dis. (2011) 11:319. 10.1186/1471-2334-11-319 22085801 PMC3226666

[15] Verver S Warren RM Munch Z Richardson M van der Spuy GD Borgdorff MW. Proportion of tuberculosis transmission that takes place in households in a high-incidence area. Lancet. (2004) 363:212–4. 10.1016/S0140-6736(03)15332-9 14738796

[16] Leonardo M Ye S Ezekiel M Allan K Philip C H Christopher C W. Transmission of *Mycobacterium Tuberculosis* in households and the community: a systematic review and meta-analysis. Am J Epidemiol. (2017) 185:1327–39. 10.1093/aje/kwx025 28982226 PMC6248487

[17] Lee MR Huang YP Kuo YT Luo CH Shih YJ Shu CC. Diabetes mellitus and latent tuberculosis infection: a *systemic* review and meta analysis. Clin Infect Dis. (2017) 64:719–27. 10.1093/cid/ciw836 27986673 PMC5399944

[18] Hensel RL Kempker RR Tapia J Oladele A Blumberg HM Magee MJ. Increased risk of latent tuberculous infection among persons with pre-diabetes and diabetes mellitus. Int J Tuberc Lung Dis. (2016) 20:71–8. 10.5588/ijtld.15.0457 26688531 PMC5652325

[19] Lin CH Kuo SC Hsieh MC Ho SY Su IJ Lin SH. Effect of diabetes mellitus on risk of latent TB infection in a high TB incidence area: a community-based study in Taiwan. BMJ Open. (2019) 9:e029948. 10.1136/bmjopen-2019-029948 31662365 PMC6830704

[20] Jackson C Southern J Lalvani A Drobniewski F Griffiths CJ Lipman M. Diabetes mellitus and latent tuberculosis infection: baseline analysis of a large UK cohort. Thorax. (2019) 74:91–4. 10.1136/thoraxjnl-2017-211124 29764958

[21] Jeon CY Murray MB. Diabetes mellitus increases the risk of active tuberculosis: a systematic review of 13 observational studies. PLoS Med. (2008) 5:e152. 10.1371/journal.pmed.0050152 18630984 PMC2459204

[22] Martinez N Kornfeld H. Diabetes and immunity to tuberculosis. Eur J Immunol. (2014) 44:617–26. 10.1002/eji.201344301 24448841 PMC4213860

[23] Marak B Kaur P Rao SR Selvaraju S. Non-communicable disease comorbidities and risk factors among tuberculosis patients, Meghalaya, India. Indian J Tuberc. (2016) 63:123–5. 10.1016/j.ijtb.2015.07.018 27451823

[24] Seegert AB Rudolf F Wejse C Neupane D. Tuberculosis and hypertension-a systematic review of the literature. Int J Infect Dis. (2017) 56:54–61. 10.1016/j.ijid.2016.12.016 28027993

[25] Caillon A Schiffrin EL. Role of inflammation and immunity in hypertension: recent epidemiological, laboratory, and clinical evidence. Curr Hypertens Rep. (2016) 18:21. 10.1007/s11906-016-0628-7 26846785

[26] Ferrannini E Cushman WC. Diabetes and hypertension: the bad companions. Lancet. (2012) 380:601–10. 10.1016/S0140-6736(12)60987-8 22883509

[27] Landsberg L Molitch M. Diabetes and hypertension: pathogenesis, prevention and treatment. Clin Exp Hypertens. (2004) 26:621–8. 10.1081/CEH-200031945 15702616

[28] Gress TW Nieto FJ Shahar E Wofford MR Brancati FL. Hypertension and antihypertensive therapy as risk factors for type 2 diabetes mellitus. Atherosclerosis risk in communities study. N Engl J Med. (2000) 342:905–12. 10.1056/NEJM200003303421301 10738048

