# Supplementary figures and images for: Prevalence of proximate risk factors of active tuberculosis in latent tuberculosis infection: A cross-sectional study from South India
Source: Front Public Health. 2022 Oct 6;10:1011388. doi: 10.3389/fpubh.2022.1011388 (PMC9583021; doi:10.3389/fpubh.2022.1011388)

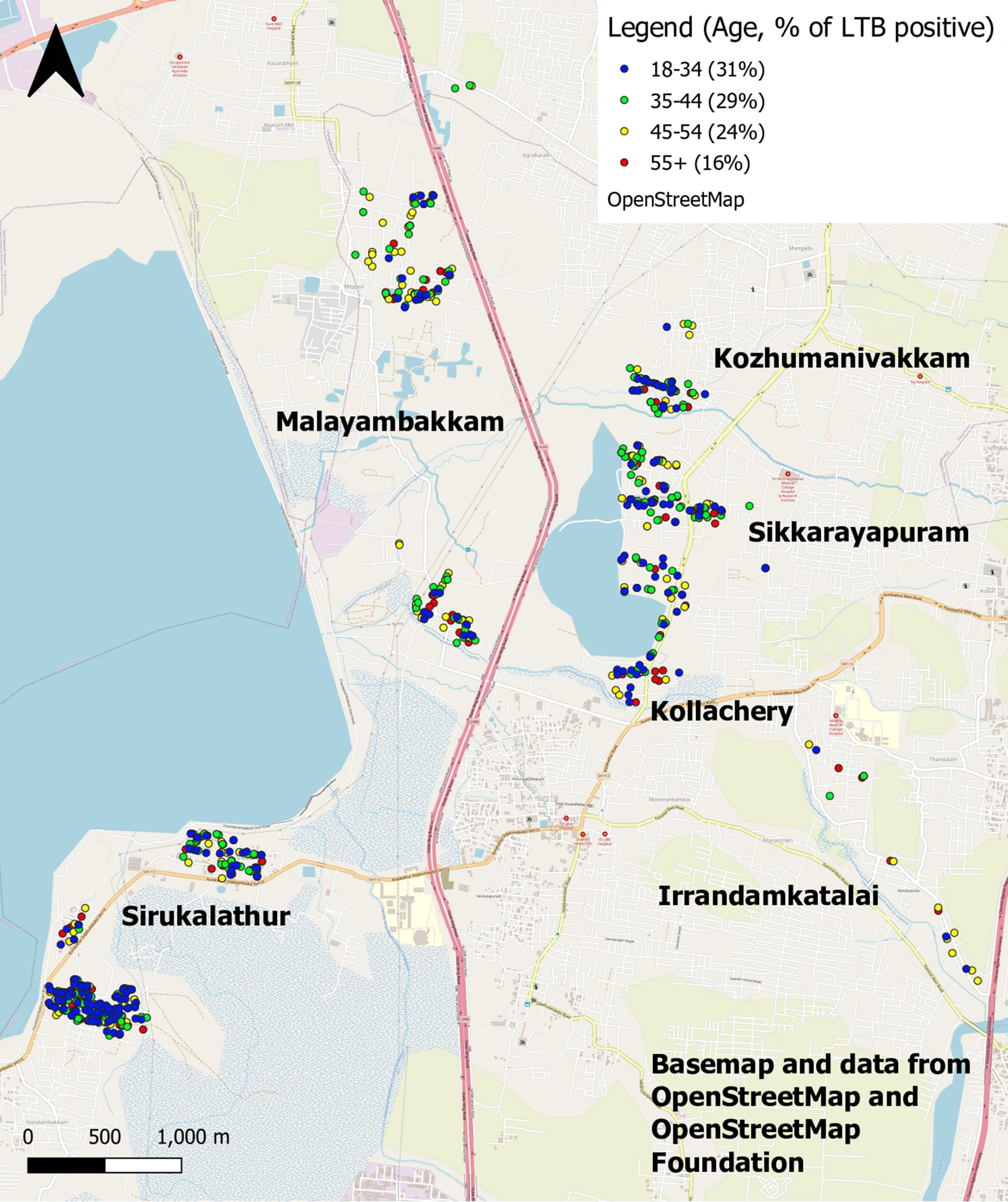

Supplement: Supplementary Figure S1 — Age-wise distribution of LTB-infected individuals. Age-wise distribution of LTB-infected individuals screened across six villages. [file Image_1.JPEG]

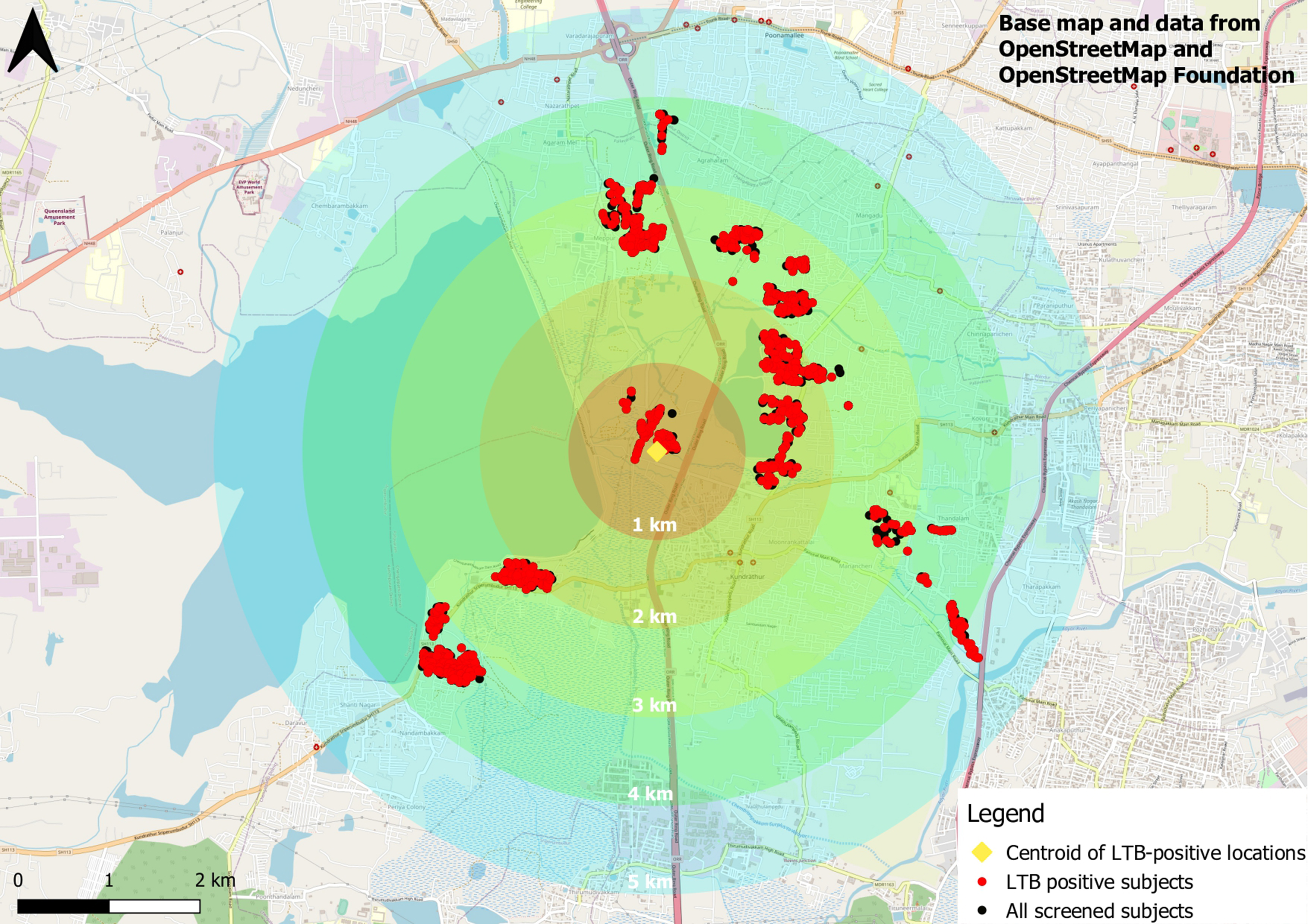

Supplement: Supplementary Figure S2 — Radius of villages screened for the prevalence of LTBI. Prevalence of LTBI screened in six villages of Kancheepuram district with 5 km radius from the center point. [file Image_2.JPEG]

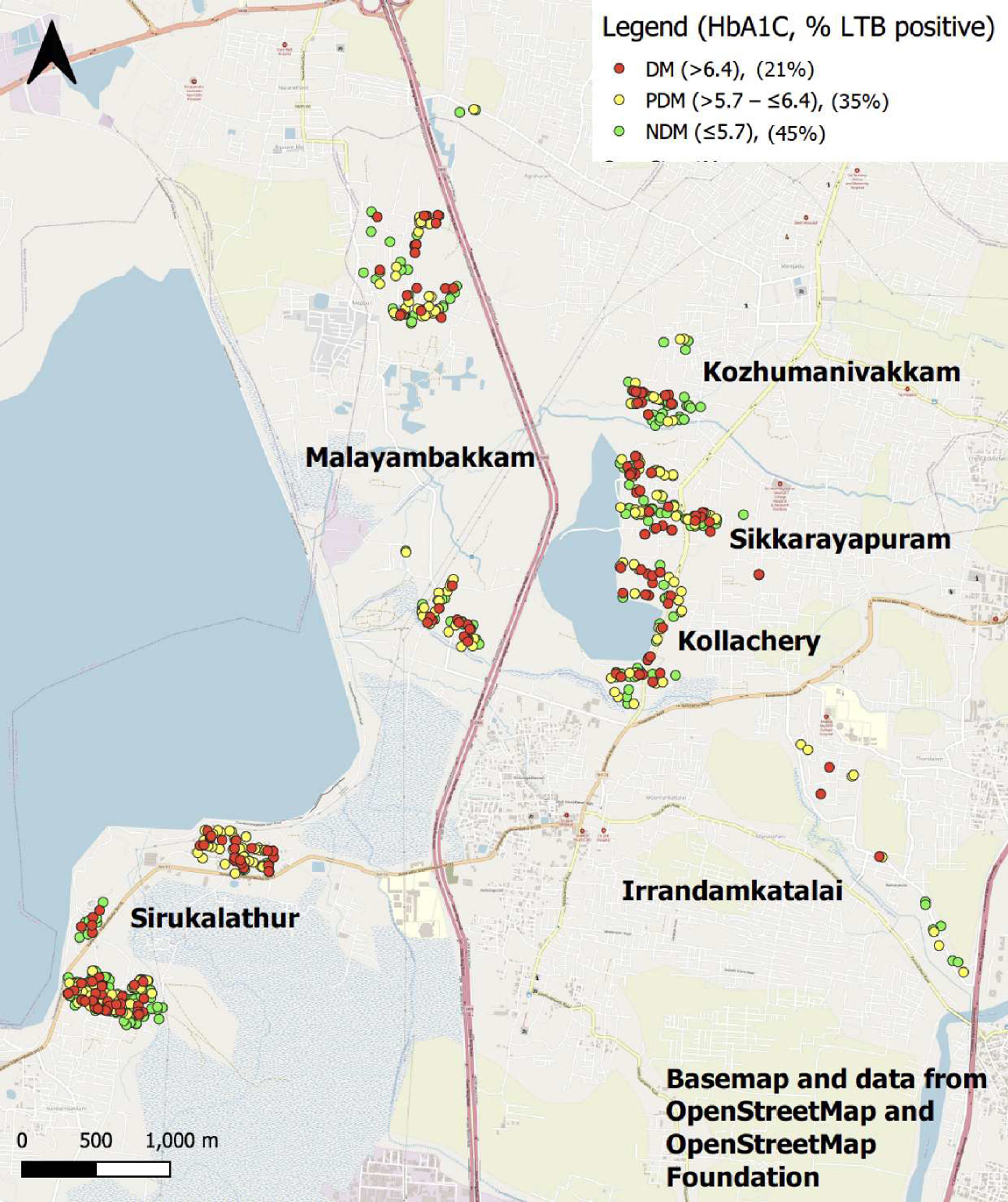

Supplement: Supplementary Figure S3 — Prevalence of diabetes. Prevalence and classification of diabetes mellitus in LTB-infected individuals screened across six villages. [file Image_3.jpg]

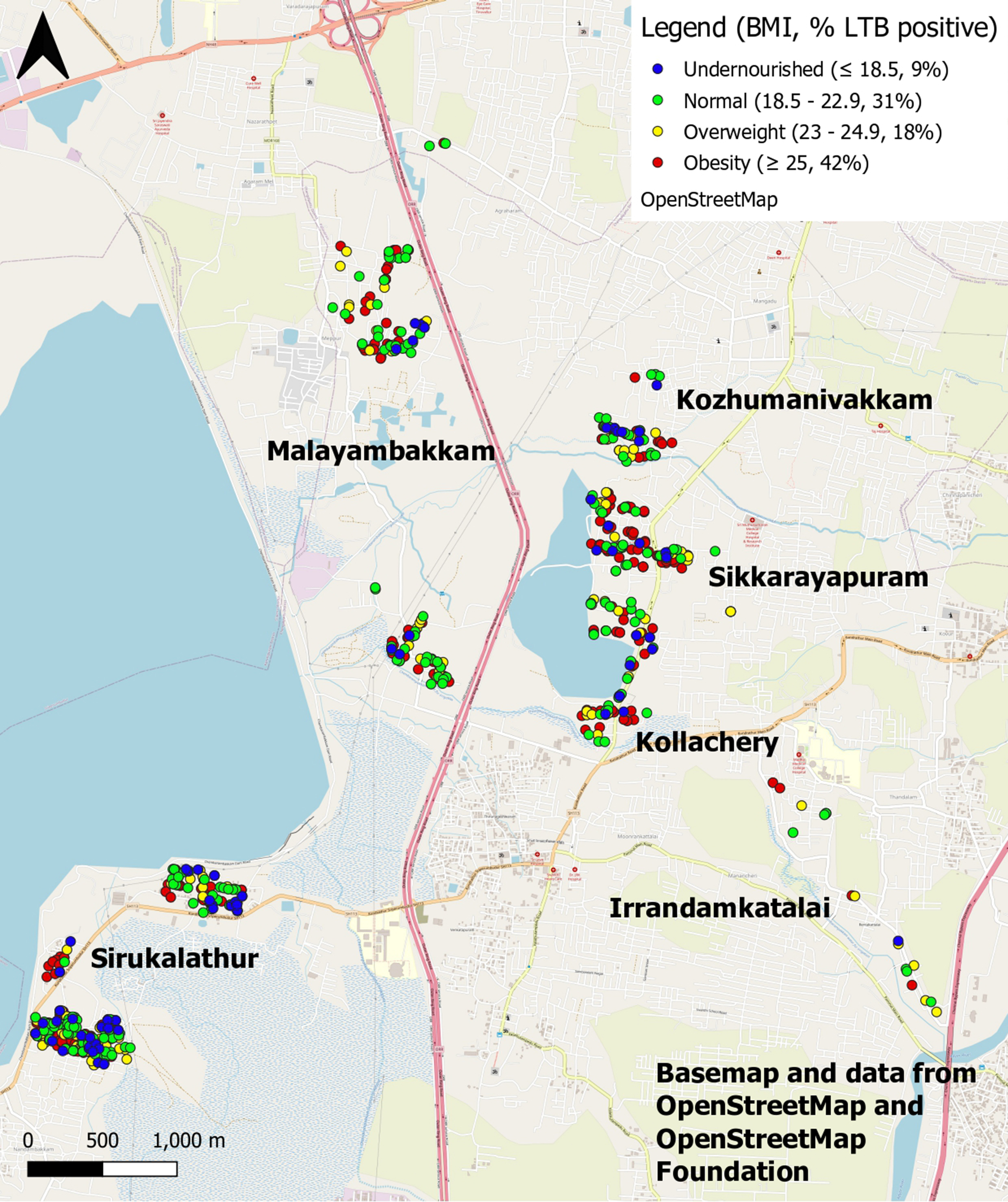

Supplement: Supplementary Figure S4 — Prevalence of BMI. Prevalence and classification of BMI in LTB-infected individuals screened across six villages. [file Image_4.JPEG]

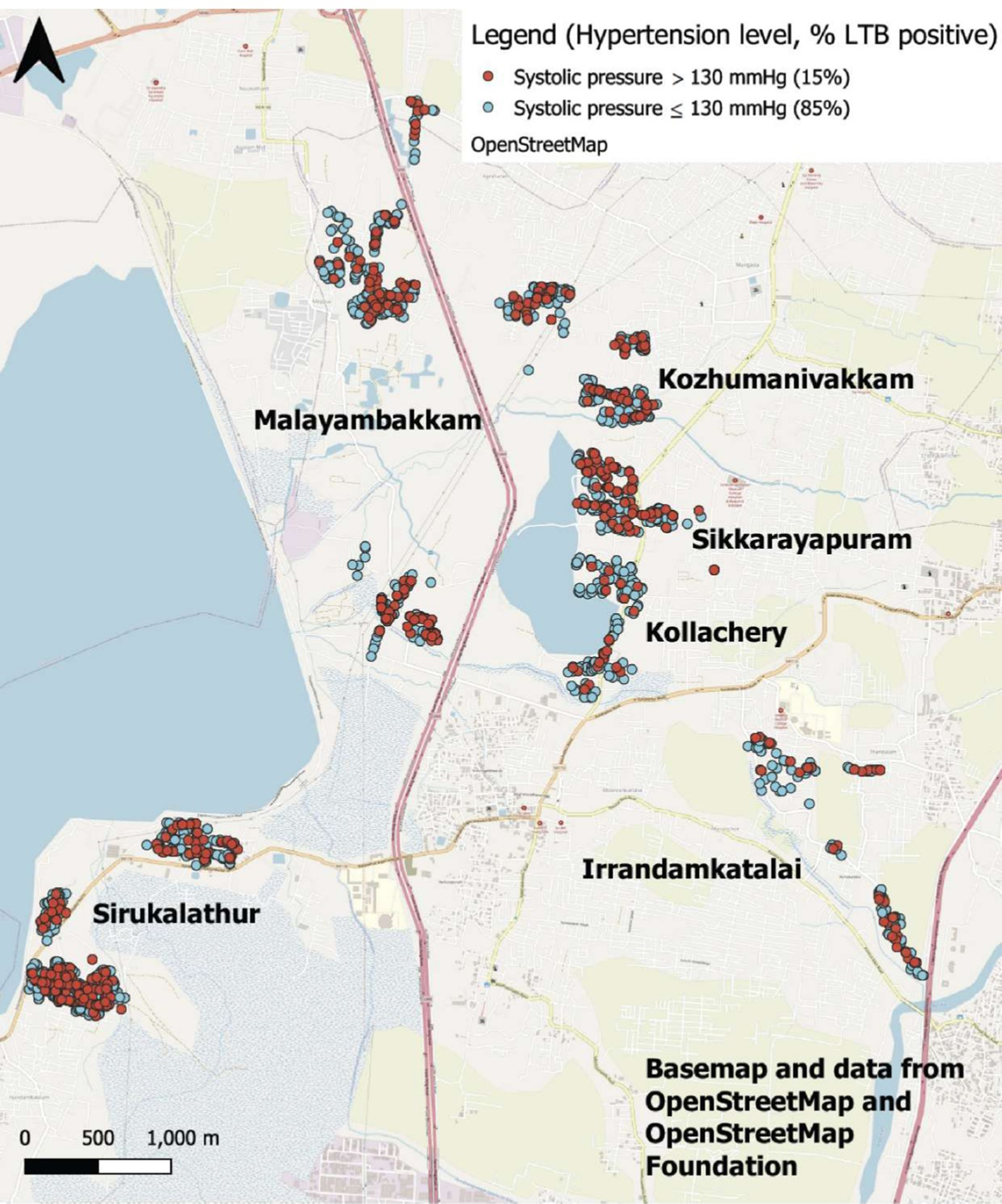

Supplement: Supplementary Figure S5 — Prevalence of HTN. Prevalence and classification of HTN in LTB-infected individuals screened across six villages. [file Image_5.JPEG]
